# Treatments with Diquat Reveal the Relationship between Protein Phosphatases (PP2A) and Oxidative Stress during Mitosis in *Arabidopsis thaliana* Root Meristems

**DOI:** 10.3390/plants13141896

**Published:** 2024-07-10

**Authors:** Adrienn Kelemen, Tamás Garda, Zoltán Kónya, Ferenc Erdődi, László Ujlaky-Nagy, Gabriella Petra Juhász, Csongor Freytag, Márta M-Hamvas, Csaba Máthé

**Affiliations:** 1 Plant Cell and Developmental Biology Research Group, Department of Botany, Faculty of Science and Technology, University of Debrecen, Egyetem sq. 1, 4032 Debrecen, Hungary; gtamas0516@gmail.com (T.G.); juhaszgabriellapetra@gmail.com (G.P.J.); freytagcsongor@gmail.com (C.F.); hamvas.marta@science.unideb.hu (M.M.-H.); 2 Department of Medical Chemistry, Faculty of Medicine, University of Debrecen, Egyetem sq. 1, 4032 Debrecen, Hungary; konya.zoltan@med.unideb.hu (Z.K.); erdodi@med.unideb.hu (F.E.); 3 Department of Biophysics and Cell Biology, Faculty of Medicine, University of Debrecen, Egyetem sq. 1, 4032 Debrecen, Hungary; lnagy@med.unideb.hu; 4 “One Health” Institute, Faculty of Health Science, University of Debrecen, Nagyerdei Blvd. 98, 4032 Debrecen, Hungary

**Keywords:** protein phosphatases, PP2A, FASS, C3-C4, histone H3 phosphorylation, mitosis, reactive oxygen species (ROS), *Arabidopsis thaliana*, diquat

## Abstract

Reversible protein phosphorylation regulates various cellular mechanisms in eukaryotes by altering the conformation, activity, localization, and stability of substrate proteins. In *Arabidopsis thaliana* root meristems, histone post-translational modifications are crucial for proper cell division, and they are also involved in oxidative stress signaling. To investigate the link between reactive oxygen species (ROS) and mitosis, we treated various Arabidopsis genotypes, including wild-types and mutants showing dysfunctional PP2A, with the ROS-inducing herbicide diquat (DQ). Studying the *c3c4* double catalytic subunit mutant and *fass* regulatory subunit mutants of PP2A provided insights into phosphorylation-dependent mitotic processes. DQ treatment reduced mitotic activity in all genotypes and caused early mitotic arrest in PP2A mutants, likely due to oxidative stress-induced damage to essential mitotic processes. DQ had a minimal effect on reversible histone H3 phosphorylation in wild-type plants but significantly decreased phospho-histone H3 levels in PP2A mutants. Following drug treatment, the phosphatase activity decreased only in the stronger phenotype mutant plants (*fass-5* and *c3c4*). Our findings demonstrate that (i) the studied PP2A loss-of-function mutants are more sensitive to increased intracellular ROS and (ii) DQ has indirect altering effects of mitotic activities and histone H3 phosphorylation. All these findings underscore the importance of PP2A in stress responses.

## 1. Introduction

Proteins undergo such post-translational modifications (PTMs) as reversible phosphorylation, which serves as a universal mechanism for regulating diverse biological functions. The phosphorylation state of proteins is a dynamic process regulated by both protein kinases and protein phosphatases. Among protein phosphatases, serine/threonine-specific phosphoprotein phosphatases (PPPs) are ubiquitous enzymes in all eukaryotes and are classified into two groups based on biochemical parameters: Type-1 (PP1) and Type-2 (PP2). Further categorization of PP2 enzymes is based on their dependency on divalent cations, resulting in PP2A, PP2B, and PP2C [1,2]. Furthermore, the PPP family includes PP4 to PP7 in a smaller amount [3]. PP2A has multiple functions in plant cells: growth and stress related signaling, including hormone-related signal transduction pathways, cell cycle regulation, vesicle trafficking, and auxin transport, as well as regulation of the activities of a huge number of enzymes involved in key metabolic pathways [3,4]. These functions occur across diverse subcellular locations, possibly due to the structural complexity of PP2A. The PP2A holoenzyme contains three subunits: an A “scaffolding” subunit, a C catalytic subunit, and a variable B regulatory subunit. These plant B subunits are further subdivided into B, B’, and B” with a wide range of molecular weights, contributing to the diverse localizations and functions of PP2A complexes [4,5].

Histone proteins are essential components of nucleosomes and targets for several PTMs, altering the structural and functional characteristics of chromatin. The DNA is wrapped around an octamer of 4 core histones: H2A, H2B, H3, H4, and fastened by the linker histone H1. Besides their globular domain, which mediates interactions among different histones within the octamer, they have a 20–35 amino-terminal residue segment that is enriched with basic amino acids and extends from the surface of the nucleosome. This residue is a target for the PTMs. The reversible phosphorylation of histones serves as a signal for transcriptional regulations and the timing of chromatin dynamics during cell division and it is involved in DNA damage repairs [6]. In plants, phosphorylation of the serine 10 and 28 residue of the N-terminal tail of histone H3 is characteristic for pericentromeric regions of chromosomes and has a crucial role in chromosome condensation, as well as being required for the proper timing of sister chromatid segregation, showing its importance in cell-cycle progression during mitosis and meiosis. A phosphorylated H3 signal first appears in late prophase, persists during metaphase and anaphase with the phosphorylation of the pericentromeric region, and disappears in telophase, when sister chromatid segregation is completed. Notably, in metaphase, the pericentromeric region shows the most intense phosphorylation signals in monocots and dicot plants (*Hordeum vulgare*, *Secale cereale*, *Ceratophyllum submersum*, and *Vicia faba*); however, protein phosphatase inhibition can cause the hyperphosphorylated state of histone H3, resulting in high phospho-histone signals at the ana- and telophase [6,7,8,9,10,11,12].

Reactive oxygen species (ROS) are normal by-products of plant metabolism, produced in several cell compartments like mitochondria, chloroplasts, and peroxisomes, as well as in the apoplast. However, under stress conditions they are produced in greater quantities. The most common are the singlet oxygen (^1^O_2_), the superoxide anion (O_2_^•−^), the hydroxyl radicals (OH^•^), and the hydrogen peroxide (H_2_O_2_) [4,13]. Oxidative stress occurs in a cell when the level of intracellular ROS exceeds the activity of scavenging mechanisms; thus, they are unable to eliminate these harmful compounds. This can cause oxidative damage of the DNA, lipid peroxidation, enzyme inactivation, and changes in the organization of microtubules, and it can trigger apoptosis [14,15,16,17]. Besides having remarkable capacities to regulate internal and external ROS sources through enzymatic and non-enzymatic scavenging processes, plant cells utilize free radicals for signaling purposes. They are involved in a variety of biological processes, such as being key regulators of plant developmental events, and even participate in hormonal as well as biotic and abiotic stress responses and the pathogen defense system. Moreover, ROS initiate signal transduction pathways; thus, they also control gene expression [13]. There is more and more evidence regarding ROS signaling in cell cycle regulation in plant cells, specifically involving cell division checkpoints [18]. Furthermore, oxidative signaling pathways also require reversible protein phosphorylation; hence, various protein kinases and phosphatases are important in the regulation of these processes [4].

Diquat (1,1′-ethylene-2,2′-bipyridylium) is a non-selective bipyridyl herbicide, structurally related to paraquat, which is used both as a contact herbicide and as a preharvest desiccant in agriculture. The herbicidal effect of these compounds relies on their redox properties. In the case of a suitable redox potential, they act as electron acceptors, thus extracting electrons from the photosynthetic electron transport system, competing with natural electron acceptors. Diquat (DQ) is easily transformed into a free radical in the redox cycle, which reacts with molecular oxygen to generate superoxide anions and, subsequently, other redox products, e.g., hydrogen peroxide. Under normal circumstances, hydrogen peroxide is neutralized by catalase and glutathione peroxidase. However, when the defense mechanisms become overwhelmed, it can induce oxidative stress and have a harmful effect on the cell. The hydroxyl radical can disrupt the lipid chains of biological membranes, initiating lipid peroxidation, and this process leads to membrane damage and eventually cell death [19]. Although these effects mainly occur in photosynthetic organs, they alter the normal growth and development of roots as well [20,21]. By affecting the mitochondrial electron transport chain and the activities of different antioxidant enzymes, DQ can cause a life-threatening poisoning in animal cells too [19,22].

The principal aim of this work is to investigate the role of specific PP2A subunits in reactive oxygen species (ROS) signaling in relation to cell cycle regulation in plants. We used the model plant *Arabidopsis thaliana* to benefit from the existing PP2A mutants as well and the wealth of data and tools available. We chose to study the role of the Arabidopsis PP2A-C3 and PP2A-C4 catalytic subunits because we have found that these two isoforms have a significant contribution to the total PP2A activity of Arabidopsis. We also decided to study the FASS regulatory subunit (B” regulatory subunit) since it has been shown to play a key role during cell division, but not much is known on its involvement in oxidative stress responses [23]. We used short-term drug treatments (24h) at different concentrations of the diquat. Analyzing protein phosphatase mutants (such as the *pp2ac3 pp2ac4* double mutant and *fass* mutant plants) treated with the oxidative stress inducer DQ enables the investigation of relationships between protein phosphatases (PP2A) and reactive oxygen species (ROS) during mitosis in *Arabidopsis thaliana* root meristems. Our main hypothesis claims that inhibiting PP2A and increasing intracellular ROS levels will impair proper cell division due to an inadequate stress response, which is typically regulated by protein phosphatases. We anticipate that this will result in (i) reduced mitotic activity and (ii) an increased level of phosphorylated histone H3 level during mitosis because of (iii) the decrease in phosphatase activity. These findings would underscore the crucial role of this enzyme in various cellular mechanisms related to the oxidative stress response and proper cell division.

## 2. Results

### 2.1. Abnormal Chromatin Organization Due to the Herbicide DQ Treatment

**Figure 1:**
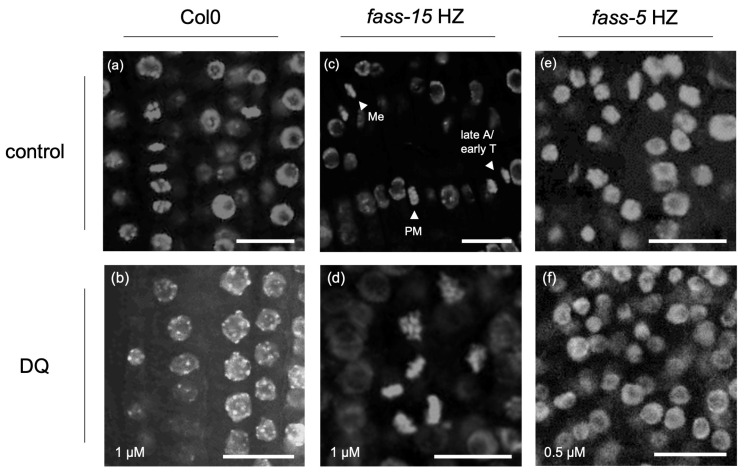
The effects of DQ on chromatin organization in non-mitotic and mitotic Arabidopsis RAM cells. (**a**,**b**) DQ inhibits mitosis and induces chromatin blebbing in Col0. (**c**,**d**) For untreated *fass-15* HZ, both early and late mitotic cells can be found (shown by arrowheads), while 1 µM DQ arrests mitotic cells in early phases. (**e**,**f**) While in untreated *fass-5* HZ, nuclear blebbing can be observed; the occurrence of blebbing decreases at DQ treatments, with smaller nucleus size. Scalebars: 5 µm. Abbreviations: PM—prometaphase; Me—metaphase; A—anaphase; T—telophase.

In non-mitotic Arabidopsis RAM cells, DQ induced chromatin blebbing (Figure 1a,b). In the case of *fass-15* HZ mutants, a mitotic arrest at the early phases can be observed, as long as we detected mitotic cells in every dividing phase in control samples; meanwhile, after 1 µM DQ treatment, the rate of the detected early mitotic phases increased dramatically (Figure 1c,d). For this genotype, slight nuclear blebbing occurred, but the occurrence did not change significantly after DQ treatment. Interestingly, in *fass-5* HZ samples, we detected decreased nuclear blebbing after DQ treatment compared to control samples, and frequent chromatin condensation also appeared, resulting in a smaller nucleus size in the interphase (Figure 1e,f). In the case of the *c3c4* double mutant, slight blebbing was detected, which was not changed dramatically by the DQ treatment. The appearance of nuclear blebbing and interphase chromatin condensation in the different genotypes after drug treatment is summarized in Table 1.

### 2.2. DQ Treatments Modulate Mitotic Activities in All Genotypes

**Figure 2:**
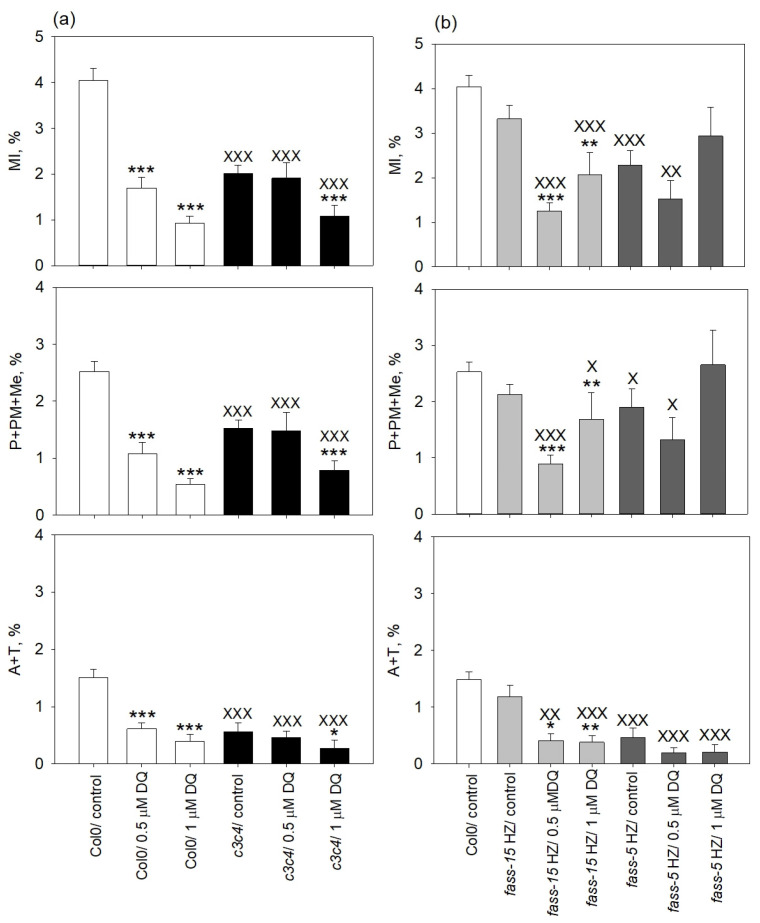
DQ has differential inhibitory effects on mitosis in RAMs of Col0 and mutants affected in PP2A subunits. (**a**) In Col0, it inhibits mitotic activity without arresting cells in any phase of mitosis. In *c3c4*, untreated cells show inhibited mitosis and slight mitotic arrest as compared to untreated Col0 cells, while *c3c4* RAMs treated with 1 µM DQ show further inhibition of mitosis. (**b**) For untreated *fass-15* and *fass-5* homozygous mutants, different degrees of mitosis inhibition can be detected, but mitosis is arrested only in *fass-5* HZ. Meanwhile, 1 µM DQ arrests cells in early mitosis in *fass-15* HZ and further amplifies mitotic arrest in *fass-5* HZ. Abbreviations: P—prophase; PM—prometaphase; Me—metaphase; A—anaphase; T—telophase. Symbols for significant differences on the graphs: X, * = significant difference (*p* < 0.05); XX, ** = significant difference (*p* < 0.01), XXX; *** = significant difference (*p* < 0.001). “X” symbols on graphs = significant differences between control wild-type (Col0) and control mutants; “*” symbols = significant differences between treatments within a given genotype.

The effects of DQ on mitotic activities were strongly genotype-dependent. For Col0, this oxidative stress inducer inhibited mitosis in a significant manner, but no mitotic arrest was observed. The concentration of 0.5 µM DQ reduced the mitotic activity by one half, and 1 µM DQ caused a slight further decrease (Figure 2a). In the case of the C catalytic subunit mutant, a slight mitotic arrest can be observed in the control (no DQ treatment) plants compared to the wild-type, but DQ treatment did not induce further change. While controls showed decreased mitosis as compared to Col0, 0.5 µM DQ did not affect the mitotic activities. A decreased mitotic index can be observed at a higher (1 µM DQ) drug concentration (Figure 2a).

For *fass-15* HZ, controls showed no significant change compared to Col0, while DQ decreased mitotic activity. Interestingly, 0.5 µM DQ inhibited mitosis to a greater extent. However, an early mitotic arrest can be observed only after 1 µM DQ treatment (Figure 2b). The *fass-5* HZ controls were characterized by decreased mitotic activities compared to Col0. However, DQ treatments slightly affected mitosis compared to the control *fass-5* HZ plants. In this mutant genotype, an early arrest also appeared, even in the control plants, and was further induced in DQ-treated RAMs compared to wild-type. In addition, 0.5 µM DQ caused a decrease in the percentage of dividing cells, and 1 µM DQ induced an apparent increase of the mitotic index, but this was due mainly to a more pronounced arrest in early mitosis, causing mitotic block, as compared to the *fass-5* HZ controls (Figure 2b).

### 2.3. Evaluation of the Phosphorylation Level of Mitotic Histone H3 in Col0 and Phosphatase Mutants and Examining the Impact of DQ Treatment

**Figure 3:**
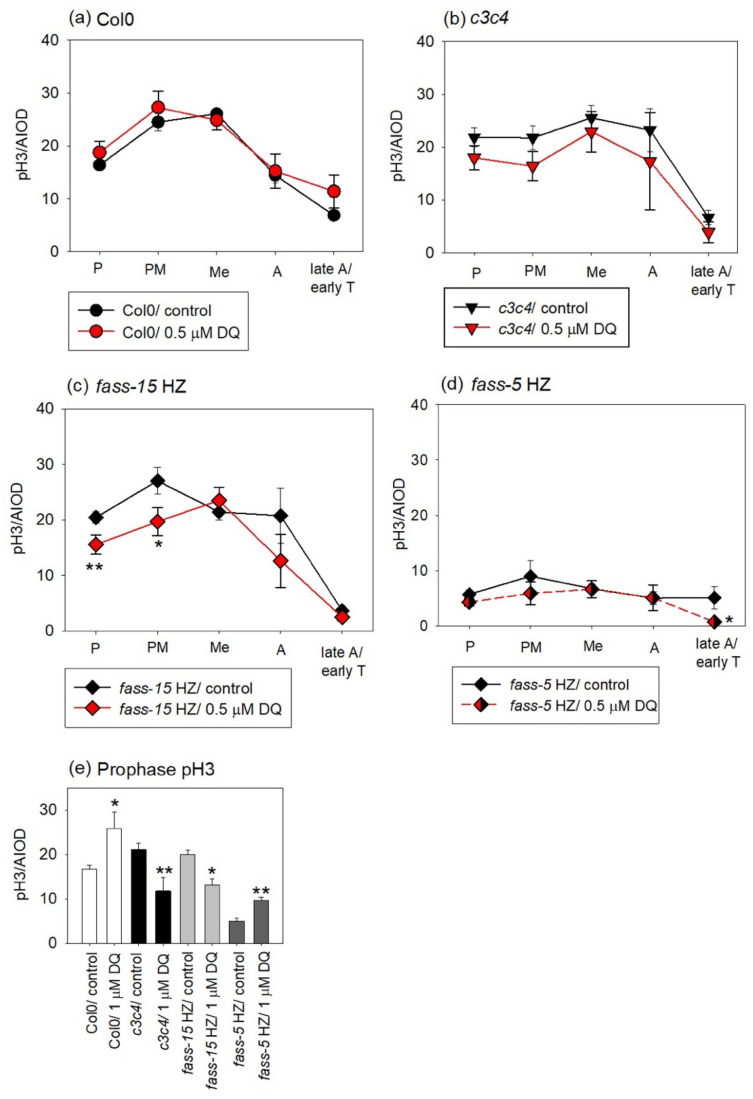
The effects of DQ on the phosphorylation state of histone H3 at serine 10 (pH3Ser10) in RAMs is genotype dependent. (**a**,**b**) DQ 0.5 μM does not induce significant changes in Col0 and *c3c4*. (**c**,**d**) DQ 0.5 μM decreases histone H3 phosphorylation; that decrease is significant for early mitotic cells in *fass-15* HZ (**c**) and late anaphase/early telophase cells in *fass*-5 HZ (**d**). (**e**) DQ 1 μM significantly increases prophase pH3Ser10 levels in Col0 and *fass-5* HZ, while it induces significant decreases in the *c3c4* and *fass-15* HZ mutants. Abbreviations: P—prophase; PM—prometaphase; Me—metaphase; A—anaphase; T—telophase. Symbols for significant differences on the graphs: * = significant difference (*p* < 0.05); ** = significant difference (*p* < 0.01); “*” symbols = significant differences between treatments within a given mitotic phase for a given genotype.

In the controls of Col0, the pH3Ser10 phosphorylation pattern was as expected for mitotic plant cells. This means that the first phospho-histone signal appears in prophase, continuously increases its level in early mitosis, and peaks in metaphase, and the dephosphorylation of histone H3 starts along with the chromosome segregation; thus, the fluorescent signal intensities decrease in late mitosis and disappear by cytokinesis. In addition, 0.5 µM DQ did not affect the phosphorylation level in Col0 (Figure 3a).

For *c3c4*, the phosphorylation of histone H3 at Ser 10 starts at a higher level compared to Col0 in prophase and increased only a little bit through the early mitotic phases. It also starts to dephosphorylate at a lower rate in the anaphase, but the pH3 signals disappear by the cytokinesis phase. Overall, DQ treatments slightly decreased the level of pH3 signals, but the phosphorylation pattern of histone H3 is like that of *c3c4* control mutants (Figure 3b).

DQ induced several notable changes in pH3Ser10 levels in *fass-15* HZ mutants. Interestingly, for the *fass-15* HZ controls, the peak of the pH3 level was in the prometaphase, and even phosphorylation started at a higher level compared to the Col0 controls. A significant decrease occurred in the early mitotic phases (prophase and prometaphase) and a non-significant decrease was observed in the anaphase after 0.5 µM DQ treatment (Figure 3c).

The most dramatic changes occurred for the *fass-5* HZ, where pH3 levels dropped significantly in control mutants compared to Col0 and to the other genotypes. In addition, 0.5 µM DQ did not affect the phosphorylation state of histone H3 compared to the *fass-5* HZ controls; only in the late anaphase could we detect a slight but significant decrease (Figure 3d).

After treatment with 1 µM DQ, we could not evaluate enough data in every mitotic phase, even after many experiments, as the division rates were too low. Here, we only show the prophase following treatment with the higher drug concentration. In this case, we could distinguish enough dividing cells and even detect significant changes. In Col0, the drug increased the pH3 level significantly. In the case of the catalytic subunit mutant (*c3c4*) and the B” regulatory subunit mutant with weaker phenotype (*fass-15* HZ), DQ decreased the phospho-histone signals almost with the same rate. For the *fass-5* HZ mutants, the level of the phosphorylated histone H3 signals increased, and almost doubled because of 1 µM DQ treatment (Figure 3e).

### 2.4. DQ Has Differential Effects on PP2A and PP1 Phosphatase Activities in Different Genotypes

**Figure 4:**
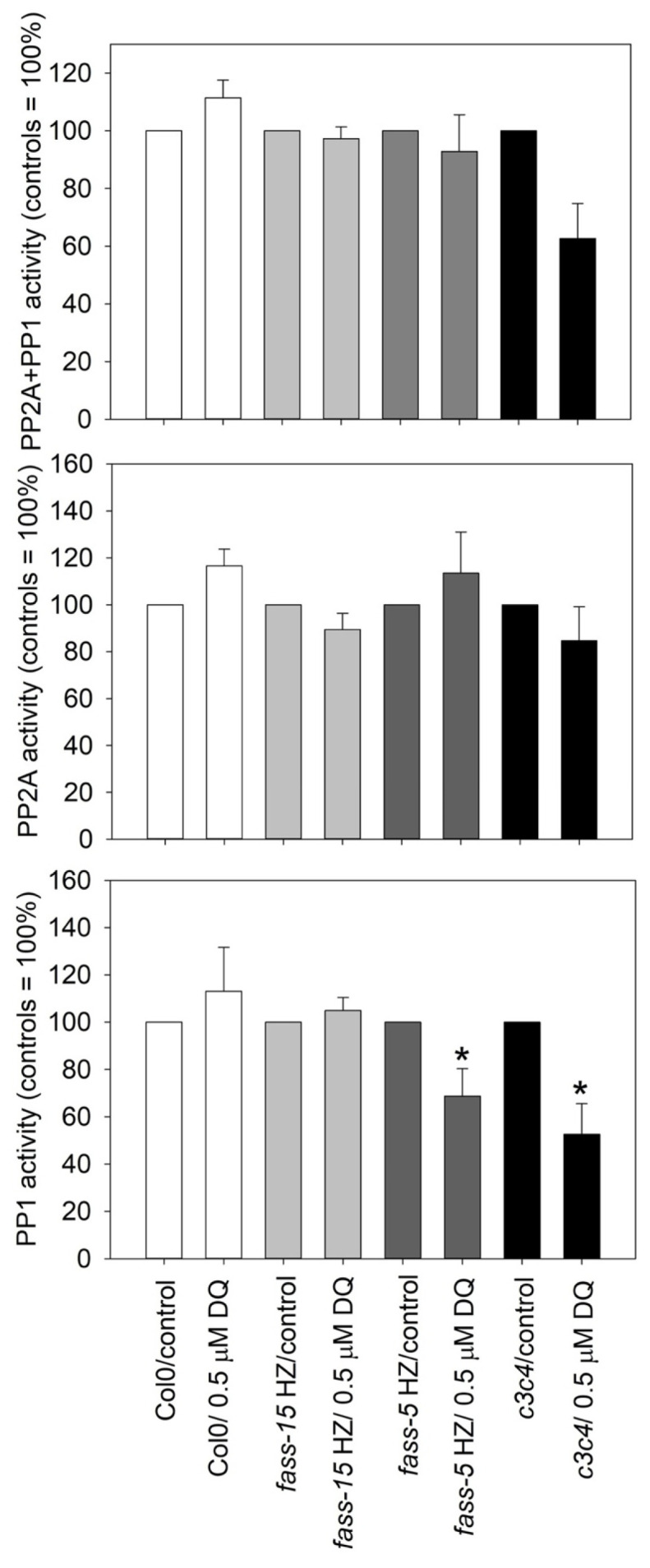
The effects of 0.5 μM DQ on total (PP2A + PP1) protein phosphatase activities as well as on separate PP2A and PP1 activities in whole seedlings of genotypes involved in this study. Significant changes (decreases) occur only in *fass-5* HZ and *c3c4* mutants for PP1. Symbols for significant differences on the graphs: * = significant difference (*p* < 0.05); “*” symbols = significant differences between treatments within a given genotype.

For protein phosphate activity assay, we only used 0.5 µM DQ treatment, as we saw from the above experiments that, at a higher concentration, the herbicide dramatically inhibits mitosis and decreases biomass, impairing the collection of samples for this type of experiment. During the evaluation of protein phosphatase activity affected by drug treatments, we took all untreated genotypes as control samples (100% phosphatase activity level). In the wild-type *A. thaliana*, the drug increased the protein phosphatase activity in total (PP2A + PP1) as well as separate PP2A and PP1 activity assays, but to a negligible extent. The *fass* HZ mutants behaved differently. In *fass-15* HZ, the drug had no effect, while in *fass-5* HZ, the drug increased the PP2A activity slightly and decreased the PP1 activity in a significant manner. In the case of the C catalytic subunit mutant, after 0.5 µM DQ treatment, a decrease in the total (PP2A + PP1) activity occurred, but this was because of the significant inhibition of PP1 activity (Figure 4).

## 3. Discussion

Programmed cell death (PCD) can be beneficial for plants during developmental processes, but ROS-induced mechanisms can initiate those pathways which eventually lead to unwanted PCD as a stress response to many abiotic factors like extreme temperatures, salinity, and pollutants, or to biotrophic pathogens [24,25]. Several hallmarks of PCD include shrinkage of the cytoplasm, condensation and aggregation of chromatin, and collapse of the plasma membrane and its separation from the cell wall, etc. [24]. We detected a major hallmark—a nuclear blebbing—after herbicide DQ treatment in wild-type plants (Figure 1a,b). Interestingly, in *fass-5* HZ mutants, nuclear blebbing was observed in control samples decreased after DQ treatment, with simultaneous inducing of chromatin condensation during interphase (Figure 1e,f). After herbicide impact, levels of reactive oxygen species (ROS) in the roots increased dramatically in *Arabidopsis thaliana* wild-type plants and mutants related to PP2A subunits. This was shown after DCFH-DA staining, which displays the total level of intracellular ROS quantity, examined by Freytag et al. [17]. Interestingly, we showed that the ROS level in control *fass-5* HZ plants was much higher than in other genotypes; however, after DQ treatment, it decreased. This result can serve as an explanation of why the occurrence of nuclear blebbing was decreasing after DQ treatment in this *fass-5* HZ mutant within 24 h.

Cell cycle regulation is sensitive to both internal and external stimuli, specifically those involving ROS. The activity of essential cell cycle proteins including cyclins and cyclin-dependent kinases (CDKs/CYCs) through redox regulation of exposed cysteine residues was directly influenced by ROS. Cell cycle arrest at key checkpoints, particularly G1/S and G2/M transition, was associated with CDK inhibition, altered cell cycle gene expression, and the activation of oxidative defense genes [18,26]. ROS also influence cell cycle exit, affecting cell expansion, the transition to the endocycle, and differentiation [18]. As we saw in our results, early mitotic arrest appeared after diquat treatments in the Arabidopsis PP2A subunit mutant samples (*fass*), but not in the wild-type (Figure 1c,d and Figure 2). The results suggested that the stronger mutation of the B” regulatory subunit mostly affected the cell cycle regulation, as an early mitotic arrest appeared also in the control *fass-5* HZ mutants. According to the previously mentioned ROS-induced regulation in the cell cycle, these early mitotic arrests can be the consequence of the increased intracellular ROS levels by diquat treatment. This stops the division at G2/M checkpoints when PP2A is dysfunctional and unable to progress with the proper cell division.

Furthermore, these early mitotic blocks can decrease mitotic activity, which we observed during the calculation of mitotic indices (Figure 2). In *Arabidopsis thaliana* wild-type plants, oxidative stress induced by diquat significantly decreased mitotic activity, without any mitotic arrest, but nuclear blebbing was observed. The sign of this PCD could be the consequence of the decreased rate of mitotic activity. Mutant plants with loss of function in specific PP2A subunits also showed reduced mitotic activity compared to Col0 controls, underscoring the importance of PP2A in cell division regulation. Notably, in the *c3c4* double mutant, only higher drug concentrations inhibited cell division; however, slight mitotic arrest at lower diquat concentrations was observed. The effects of DQ on homozygous B” regulatory subunit mutants varied with concentration and due to the strength of the mutation, indicating a significant role of the B” subunit in the oxidative stress response.

In addition, the herbicide had distinct inhibitory effects on mitotic activity, as well as inducing early mitotic arrest in *fass* heterozygous mutant plants (Supplementary Figure S1) compared to Col0 at different DQ concentrations. This suggests that despite being the same phenotype as the wild type, dysregulation in the cell cycle at the cellular level occurred after mutation, further suggesting PP2A’s involvement in mitotic regulation.

Epigenetic mechanisms play critical roles during the life cycle; these include several PTMs, like histone modifications, or, even more, histone variants that regulate the structure and accessibility of chromatin and thereby impact the chromatin’s biological function [27]. It has been documented that pathogen attack and diverse abiotic stresses significantly modify epigenetic markers [28]. Reversible epigenetic modifications include methylation, acetylation, ubiquitination, or phosphorylation at histone tails, which can lead to the silencing of a previously active gene or to the activation of a silent genetic region [29].

Several studies have observed the induction of H3 phosphorylation in response to abiotic stress. Here are a few examples: In *A. thaliana* and tobacco cells, an increase in phosphorylated histone H3 level at serine 10 was detected shortly after exposure to high salt concentrations [30]. In *V. faba*, there is also evidence for the involvement of Aurora B kinase-mediated phosphorylation of serine 10 on histone H3 (pH3Ser10) under heavy metal stress modifications and responses [31]. Hydroxyurea (HU)-treated *Allium cepa* cells demonstrated that additional sites of H3Ser10 phosphorylation are induced under stress conditions at sites of chromosomal breakage due to DNA damage, and pH3Ser10 signals were shown also in the G2-phase and in late telophase [32]. Cold treatment induces H3 phosphorylation at the nucleolar organizer region, heterochromatic sites, and telomeres. Moreover, ice-water treatment of meristems resulted in additional sites of H3 phosphorylation in metaphase chromosomes besides the centromere of barley, rye, and *V. faba* [12].

After herbicide treatment, we also detected various changes in the phosphorylation level of histone H3 at serine 10. After drug exposure for 24 h at lower DQ concentration, mutants impaired in PP2A subunits were more affected than the wild-type plants (Figure 3a–d). Overall, a slight decrease can be observed in every mutant genotype in different mitotic phases (Figure 3b–d). Higher drug concentration strongly inhibited the activity of cell division, as shown by the mitotic indices (Figure 2), resulting in fewer dividing cells, with a different effect on the phospho-histone H3 level in the examined genotypes (Figure 3e). Most interestingly, in *fass-5* HZ mutants, a very low pH3 level was observed, which further decreased after lower herbicide concentrations and increased at higher DQ impacts during prophase. In addition, higher herbicide concentrations increased pH3 levels in prophase in Col0, too. However, in *fass* HeZ mutants, the DQ treatment increased the phosphorylation level of histone H3 in a significant manner in different mitotic phases compared to Col0 and *fass* homozygous mutant plants (Supplementary Figure S2). This suggests that PP2A plays a role in oxidative stress-induced regulations during mitosis, and its lack or decrease of function induces changes in the phosphorylation-dependent regulation processes.

It is worth mentioning that plants also contain a distinct subclass of variants of core histone proteins that are stress inducible. For example, the *A. thaliana* genome encodes the canonical H2A and its variants, which are involved in stress response systems [30]. γH2A.X, the phosphorylated form of H2A.X variant, occurs rapidly after double-stranded DNA damage by oxidative stress and plays a key role in the recruitment of DNA repair proteins [17,30], in which PP2A has a curial role, proved by Freytag et al. [17]. An increased level of ROS after DQ treatment exceeds cell homeostasis and impacts different cellular processes, such as the inducing of γH2A.X formation or the modulation of key enzymes (SOD; POD) involved in ROS scavenging in Arabidopsis. The direct or indirect involvement of B” and C3/C4 subunits of PP2A in the regulation of ROS and γH2A.X levels and of SOD activities was proven [17]. Most interestingly, in *fass-5* HZ, DQ treatment decreased the level of γH2A.X dramatically, instead of increasing as we expected. This can correlate to the fact that when a stronger chromatin condensation occurred after drug treatment, the nucleosome was closed to PTMs, resulting in the decreased level of γH2A.X, and the oxidative stress had already initiated the PCD event (nuclear blebbing and chromosome condensation). Superoxide dismutase (SOD) activity assays reveal that both the B” and C subunits of PP2A modulate SOD activities and DQ increase their activities in a significant manner, resulting in the induction of the ROS scavenging mechanism [17]. It is worth mentioning that, for *c3c4*, 1 µM DQ induced decreases in total peroxidase (POD) activity, in spite of Col0, where we observed increases. This indicates that, in *c3c4*, ROS scavenging mechanisms may be impaired at increased oxidative stress.

ROS are known to modulate functioning of protein kinases and phosphatases, leading to the regulation of several phosphorylation-dependent signaling cascades and certain gene expressions [33]. Most of the studies deal with the involvement of the PP2C in plant stress signals and responses, which play a curial role in drought stress, while ABA, high salinity, low temperature, and osmotic stress also trigger the upregulation of PP2C genes [34]. PP1 is involved in plant–pathogen interactions and, indeed, regulating plant immune responses [35]. In the abiotic stress response, expression analysis of PP1 in soybean revealed the role of this enzyme in drought tolerance [36] and the involvement of *Oryza sativa* protein phosphatase 1a (OsPP1a) in salt stress tolerance in transgenic rice [37]. The involvement of various B regulatory subunits of PP2A in plant development and stress reactions is summarized by Máthé et al. [4,38]. The C catalytic subunit can be involved in both the biotic and abiotic stress responses, including ROS scavenging and DNA damage repair with the interaction of B” regulatory subunit [17,38].

For Col0, we revealed that oxidative stress significantly inhibited mitosis without altering phospho-histone H3 levels or protein phosphatase activity. In *fass-15* HZ, diquat treatments caused a significant reduction in the pH3 level and mitotic arrest but without any changes in protein phosphatase activity, like in Col0. In the case of *fass-5* HZ, a significant inhibition after DQ treatment can be only observed in the PP1 activity, along with the low pH3 levels. Meanwhile, the total protein phosphatase activities (PP2A + PP1) were affected by the drug treatment in *c3c4* mutant plants; however, only PP1 activity was inhibited significantly (Figure 4) with the decrease of pH3 level. These data indicate that in more pronounced PP2A mutant phenotypes, the protein phosphatase PP1 is more sensitive to oxidative stress.

## 4. Materials and Methods

### 4.1. Plant Material and DQ Treatments

The genotypes of *Arabidopsis thaliana* used in this study were the wild-type Columbia ecotype (Col0) as well as protein phosphatase (PP2A)-related mutants described by Camilleri et al., Kirik et al., Ballesteros et. al., and Spinner et al. [23,39,40,41]. In the case of *fass-5* [39] and *fass-15* [40] mutants, the function of the regulatory subunit B” is lost, while in the case of *c3c4* double mutant [41,42], the function of the catalytic subunit is impaired. The catalytic subunit mutant is designed by a T-DNA insertional mutagenesis by *Agrobacterium tumefaciens*, and the FASS mutants were created by ethyl methanesulfonate (EMS) mutagenesis. The *c3c4* double mutants and the *fass* homozygote recessive (HZ) phenotypes show severe developmental disorders: dwarfism, defects in the development of axial organs, and lack of preprophase band (PPB) in the meristematic cells. *fass-5* HZ is a strongly altered phenotype with the lack of fully differentiated axial organs [23]. In the case of loss-of function mutants for the B” regulatory subunit, we distinguish a weaker (*fass-15* HZ) and a stronger (*fass-5* HZ) mutant phenotype, which appears in the state of development. The *fass* heterozygotes (HeZ) are showing apparently normal phenotypes. In general, HeZs were, in fact, pools of seedlings consisting of the homozygote dominant genotype (in fact, Col0) and *fass* HeZs plants. These pools are referred to hereafter as heterozygotes, as, phenotypically, HeZs cannot be distinguished from wild-type seedlings and the results for them are presented as supplementary data.

Seed sterilization, seedling culture, and the method for drug treatments were performed essentially according to Freytag et al. [17]. Seeds’ surface was sterilized with commercial bleach and they were transferred to a Murashige-Skoog medium with Gamborg’s vitamins (MS*), containing 2% sucrose [43,44]. After 48 h of cold treatment, plates with seeds were transferred to a plant tissue culture chamber under a 14/10 h photoperiod, 22 ± 2 °C, 60 µmol m^−2^s^−1^ photon flux density in the light period. Drug treatment started with five-days old seedlings, by transferring them on sterilized filter paper soaked in liquid MS* media (for control) and 0.5 and 1 µM diquat (DQ, Supelco Inc., Bellefonte, PA, USA) solution (diluted in liquid MS* media). For this study, we selected DQ concentrations that were non-lethal over a 24 h treatment period but still effective in inducing oxidative stress responses.

We employed primary roots for all Arabidopsis genotypes in our immunohistochemistry and histochemistry studies. However, due to poorly developed or sparse root biomass, we utilized whole seedlings for the *fass* HZ and *c3c4* mutants along with the control Col0 for protein phosphatase activity assays.

### 4.2. Immunofluorescence and Histochemical Labeling Procedures

After drug treatment, phosphorylated histone H3 (pH3Ser10), microtubules (β-tubulin), and chromatin were labeled by immunohistochemical and histochemical methods. Whole-mount labeling protocol was used to examine the organization of the specific antigens inside the plant cell. The main procedure was as described by Pasternak et al. [45]: fixation, permeabilization, blocking, and addition of primary and secondary antibodies.

The plants were fixed with 2% (*w*/*v*) paraformaldehyde (PFA) + 0.1% (*v*/*v*) Triton X-100 in 2× microtubule stabilizing buffer (MTSB). After 2× 5 min of vacuum infiltration, the samples were further incubated in the solution for 50 min without vacuum. Then the samples were washed 3 times with 1× MTSB. Root tips were treated with methanol for 10 min and rehydrated with a series of decreasing methanol concentrations in every 3 min. The samples were washed 3 times with ultrapure water. Afterwards, cell walls were digested with 0.2% (*w*/*v*) Driselase (Sigma-Aldrich, St. Louis, MO, USA) and 0.15% (*w*/*v*) Macerozyme (Serva Electrophoresis GmbH, Heidelberg, Germany), dissolved in 2 mM MES (Sigma-Aldrich, St. Louis, MO, USA), for 25 min at 37 °C and samples were washed with 1× MTSB three times. Seedlings were then permeabilized with 10% (*v*/*v*) DMSO + 3% (*v*/*v*) IGEPAL (Sigma-Aldrich, St. Louis, MO, USA) in 1× MTSB for 20 min at 37 °C and washed with 1× MTSB three times. Pre-incubation was performed with 4% (*w*/*v*) BSA (Sigma-Aldrich, St. Louis, MO, USA) in 1× MTSB for 30 min. After that, primary antibodies were diluted in 4% (*w*/*v*) BSA + 1× MTSB, and samples were incubated overnight in 4 °C, then washed with 1× MTSB three times. The primary antibodies were anti-β-tubulin raised in rabbit (Abcam, Cambridge, UK) at a 1:100 dilution and anti-histone H3 (anti-phospho-H3 at serine 10) raised in rabbit (Abcam, Cambridge, UK) at a 1:50 dilution. The two immunolabeling procedures were performed in separate experiments, because of the same host immunogen animal. Secondary antibody was Alexa 488 conjugated anti-rabbit IgG generated in goat (Abcam, Cambridge, UK) at a 1:200 dilution for microtubule labeling and at a 1:100 dilution for phospho-histone H3 labeling. Incubation with secondary antibody was in 4% (*w*/*v*) BSA + 1× MTSB for 4 h at 37 °C, then samples were washed with 1× MTSB three times. Thereafter, a fluorescent dye, 4′6′-diamidino-2-phenylindole (DAPI; Fluka, Buchs, Switzerland) was used to visualize all nuclei. The samples were incubated with 3 µg mL^−1^ DAPI diluted in 1× MTSB for 30 min, then washed 3 times with 1× MTSB. Finally, sample preparation followed. We fixed the plants on the slide using Mowiol^®^ 4-88 (Sigma-Aldrich, St. Louis, MO, USA) as a mounting media.

The fluorescent labeled samples analyzed by confocal laser scanning microscopy (CLSM). Where microtubules and chromatin were labeled, the samples were visualized with Zeiss LSM 880 (Carl Zeiss AG, Jena, Germany) confocal microscope with Zen Black 2.3 software. Conventional settings for Alexa 488 visualization: Arg laser for excitation; emission was observed beside a 490 nm dichroic mirror and a 490–530 filter set. DAPI visualization was performed with a 405 nm diode laser and signal detection was as for the conventional LSM parameters for this dye. Samples with phospho-histone H3 and chromatin labeling were visualized with a Nikon Ti-E inverted super-resolution microscope (Nikon Instruments Inc., Melville, NY, USA) with NIS-elements Ar software (version number: 5.30.02) and we used Plan Apo VC 60×A WI DIC N2 objective. For DAPI dye visualization we used 405 nm laser with 405/50 filter set and the emission wavelength was 425/475. For the detection of Alexa 488 dye we used 488 nm laser and 525/50 filter set. Emission was observed at the wavelength of 500/550 in this case.

### 4.3. The Quantification of Mitotic Activities and Histone H3 Phosphorylation Signal

Mitotic indices (MI) were calculated as the percentage of total mitotic cells, and separately as the percentages of early (prophase, prometaphase, and metaphase) and late (anaphase, telophase, and cytokinesis) mitotic cells, out of the total cell number in the root apical meristems (RAMs) of primary roots. To ensure accurate results, we used separate methods for quantifying mitotic indices: microtubule labeling, which provided a clearer distinction of late mitotic phases, and phosphorylated histone H3 labeling, which improved differentiation of early mitotic phases. The quiescent center zone and the meristematic cells destined to form vascular tissue and the root cap region were excluded from the counts. We quantified the pH3Ser10 fluorescent signal intensity using Fiji software version 2.0.0. [46]. This was expressed as the number of pixels showing area integrated optical density (AIOD) after background extraction. Roots from at least three seedlings per treatment per experiment were analyzed, and a minimum of five independent experiments were conducted.

### 4.4. The Assay of Protein Phosphatase Activities

Protein phosphatase assays were conducted following established protocols [15,47,48] to measure total protein phosphatase (sum of PP2A and PP1) as well as PP2A and PP1 activities separately. Plant extracts were prepared using a buffer containing 50 mM Tris-HCl (pH 7.5), 0.1 mM EDTA, 0.2 mM EGTA, 0.1% (*w*/*v*) DTT, 1 mM phenylmethylsulfonyl fluoride (PMSF; Sigma-Aldrich, St. Louis, MO, USA), and 0.15% (*v*/*v*) protease inhibitor cocktail (Roche Applied Science, Indianapolis, IN, USA), then homogenized on ice. After centrifugation at 4 °C, the supernatant’s protein content was assayed using the Bradford method [49]. The substrate for the phosphatase assay was ^32^P-MLC20 (turkey gizzard 20 kDa myosin-light chain). We used phosphorylated myosin light chain from smooth muscle as a substrate because it is primarily dephosphorylated by both PP1 and PP2A in cell extracts. The assay mixture included the supernatant containing 10 µg protein of cell lysates and 2 µM [^32^P]-myosin-light chain substrate. Specific protein phosphatase activities were expressed as pmol ^32^Pi released mg protein^−1^. To distinguish PP2A from PP1 activities, 2 µM Inhibitor-2 (I-2), a natural PP1 inhibitor, was added. PP2A activity was measured from the extracts after adding I-2, while PP1 activity was determined by subtracting PP2A activity from the total protein phosphatase activity [50]. All activity assays were measured in 3–6 repetitions per plant sample, with control seedling extract activity set at 100%.

### 4.5. Data Analysis

All quantified data were visualized with the aid of Systat Sigma Plot 10.0^®^ and 12.0^®^ softwares (Systat Software, San Jose, CA, USA) and diagrams are showing the mean ± SE values. “X” symbols on graphs = significant differences between control wild-type (Col0) and control mutants; “*” symbols = significant differences between treatments within a given genotype. Statistical significances for the differences between controls and treatments were studied by two-way ANOVA (post-hoc: Holm-Sidak method) and t-tests. Symbols for significant differences on the graphs: X, * = significant difference (*p* < 0.05); XX, ** = significant difference (*p* < 0.01), XXX; *** = significant difference (*p* < 0.001).

## 5. Conclusions

When the PP2A holoenzyme was dysfunctional in its different subunits, oxidative stress induced various responses, depending on whether the C catalytic subunit or the B” regulatory subunit function was inhibited. Oxidative stress induced cell death signals, such as nuclear blebbing and increased chromatin condensation, in a genotype-dependent manner. A frequent blebbing occurred in wild-type plants after drug treatment with strongly inhibited mitotic activity, but arrest did not appear in wild-type plants. In the case of *fass-5* HZ mutants, a complete chromatin condensation enhances PCD after oxidative stress induction. Additionally, DQ induced mitotic arrest in early mitosis in PP2A mutant plants, together with the common strong mitotic activity reduction. Overall, these observations were not linked to histone H3 phosphorylation, indicating, rather, a general response to oxidative stress. DQ did not affect PP2A activity but it decreased PP1 activity in stronger phenotype mutants (*fass-5* HZ and *c3c4*). Despite our expectations, we observed various cellular effects after oxidative stress induction and alteration of PP2A functionality, from which we are suggesting only an indirect correlation between PP2A and ROS-induced histone H3 phosphorylation changes. The lack of consistently higher pH3 levels under the influence of DQ may imply a potential direct interaction with Aurora B kinase, responsible for histone H3 phosphorylation. Further investigation is needed to confirm this interaction and it will be the focus of future research. These data lead us to conclude that the PP2A holoenzyme, possibly by the crosstalk with PP1, indirectly regulates proper cell cycle regulation under oxidative stress.

## Figures and Tables

**Table 1:** Occurrence of nuclear blebbing and/or interphase chromatin condensation in the RAMs of primary roots; rounded % of total RAMs.

	Col0	*fass-15* HZ	*fass-5* HZ	*c3c4*
**control**	6%	31.5%	35%	16%
	slight blebbing	slight or frequent blebbing, sporadic chromatin condensation	mostly slight blebbing, frequent chromatin condensation	slight blebbing, some chromatin condensation
**0.5 μM DQ**	35%	33.5%	7%	22%
	slight or frequent blebbing	mostly slight blebbing, sporadic chromatin condensation	slight blebbing, frequent chromatin condensation	slight or frequent blebbing
**1 μM DQ**	33.5%	50%	11%	14%
	slight blebbing	mostly slight blebbing	slight blebbing, frequent chromatin condensation	slight blebbing

## Data Availability

All relevant data can be found within the manuscript.

## Supplementary Materials

The following supporting information can be downloaded at: https://www.mdpi.com/article/10.3390/plants13141896/s1, Supplementary Figure S1: The effects of DQ on mitotic activities of RAMs in heterozygote *fass* genotypes; Supplementary Figure S2: The effects of DQ on pH3Ser10 levels in RAMs of heterozygote *fass* genotypes.
