# Supplementary material for: Treatments with Diquat Reveal the Relationship between Protein Phosphatases (PP2A) and Oxidative Stress during Mitosis in Arabidopsis thaliana Root Meristems
Source: Plants (Basel). 2024 Jul 10;13(14):1896. doi: 10.3390/plants13141896 (PMC11279869; doi:10.3390/plants13141896)
Supplement: Supplementary file 1 [file plants-13-01896-s001.zip › plants-3055366-supplementary.pdf]

Supporting Information

Article title: Treatments with diquat reveal the relationship between protein phosphatases (PP2A) and oxidative stress during mitosis in Arabidopsis thaliana root meristems

Authors: Adrienn Kelemen, Tamás Garda, Zoltán Kónya, Ferenc Erdődi, László Ujlaky-Nagy, Gabriella Petra Juhász, Csongor Freytag, Márta M-Hamvas, Csaba Máthé

The following Supporting Information is available for this article:

Supplementary Figure S1 The effects of DQ on mitotic activities of RAMs in heterozygote *fass* genotypes

Supplementary Figure S2 The effects of DQ on pH3Ser10 levels in RAMs of heterozygote *fass* genotypes

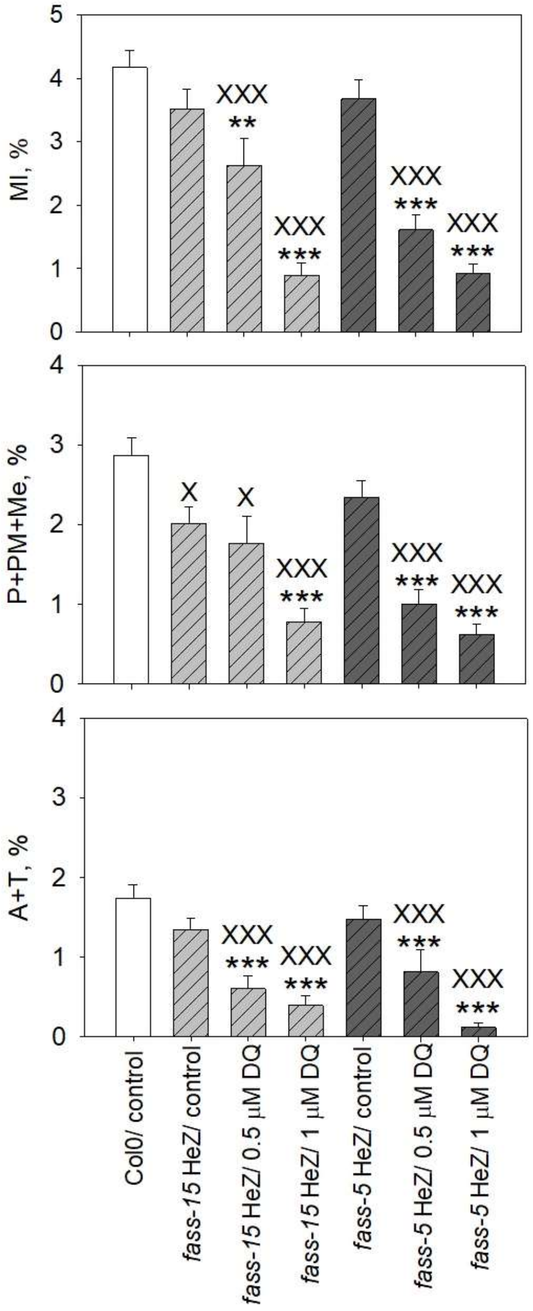

Supplementary Figure S1. The effects of DQ on mitotic activities of RAMs in heterozygote *fass* genotypes. DQ has inhibitory effects, but in contrast to Col0, where no arrests can be detected (see Figure 2), 0.5 µM DQ induces early mitotic arrest in *fass-15* HeZ and 1 µM DQ induces early mitotic arrest in *fass-5* HeZ. Abbreviations: P – prophase; PM – prometaphase; Me – metaphase; A – anaphase; T – telophase. Symbols for significant differences on the graphs: X, * = significant difference ($P < 0.05$); XX, ** = significant difference ($P < 0.01$), XXX; *** =significant difference ($P < 0.001$). “X” symbols on graphs = significant differences between control wild-type (Col0) and control mutants; “*” symbols = significant differences between treatments within a given genotype.

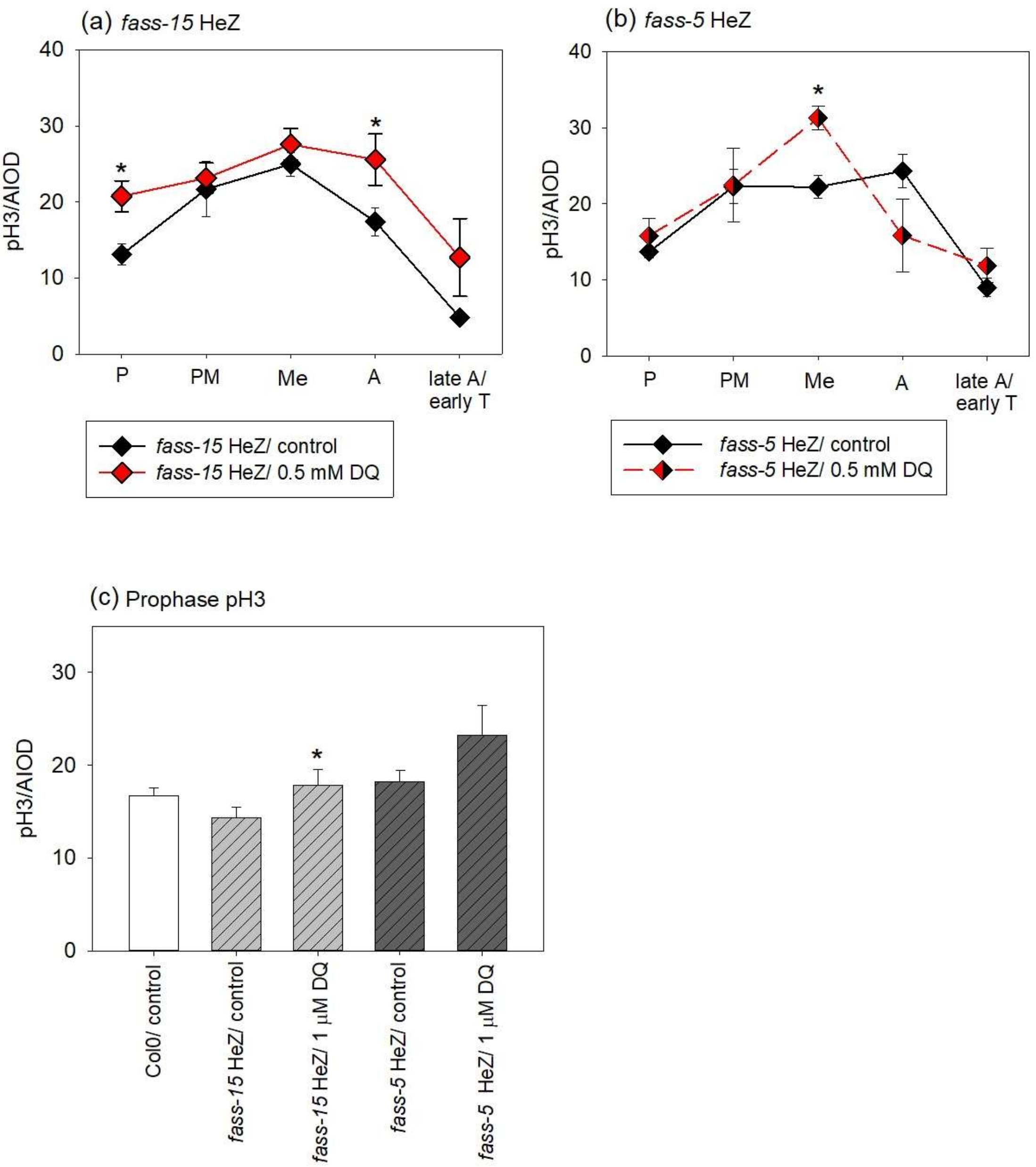

Supplementary Figure S2. The effects of DQ on pH3Ser10 levels in RAMs of heterozygote *fass* genotypes. (**a, b**) In contrast to Col0, where 0.5 µM DQ does not induce significant changes and to *fass* homozygotes, where it decreases pH3 levels (see Figure 3), in these heterozygotes DQ induces significant increases in several mitotic phases. (**c**) 1 µM DQ increases prophase pH3Ser10 levels, as for Col0 (see Figure 3). Abbreviations: P – prophase; PM – prometaphase; Me – metaphase; A – anaphase; T – telophase. Symbols for significant differences on the graphs: * = significant difference (P < 0.05); "*" symbols = significant differences between treatments within a given genotype.
